# Assessing colposcopy competencies in medically underserved communities: a multi-center study in China

**DOI:** 10.1186/s12885-024-12106-y

**Published:** 2024-03-19

**Authors:** Xiaoli Cui, Huike Wang, Mingyang Chen, Samuel Seery, Peng Xue, Youlin Qiao, Yuhong Shang

**Affiliations:** 1 Dalian Medical University, Dalian, Liaoning 116044 China; 2 Department of Gynecologic Oncology, Cancer Hospital of China Medical University, Liaoning Cancer Hospital & Institute, Shenyang, Liaoning, 110042 China; 3 School of Population Medicine and Public Health, Chinese Academy of Medical Sciences and Peking Union Medical College, Beijing, 100005 China; 4 Faculty of Health and Medicine, Division of Health Research, Lancaster University, Lancaster, LA1 4YW UK; 5 Department of Gynecology, First Affiliated Hospital of Dalian Medical University, Dalian, Liaoning 116021 China

**Keywords:** Colposcopy, Health workforce, Competence, Underserved communities

## Abstract

**Background:**

Colposcopy plays an essential role in diagnosing cervical lesions and directing biopsy; however, there are few studies of the capabilities of colposcopists in medically underserved communities in China. This study aims to fill this gap by assessing colposcopists’ competencies in medically underserved communities of China.

**Methods:**

Colposcopists in medically underserved communities across China were considered eligible to participate. Assessments involved presenting participants with 20 cases, each consisting of several images and various indications. Participants were asked to determine transformation zone (TZ) type, colposcopic diagnoses and to decide whether biopsy was necessary. Participants are categorized according to the number of colposcopic examinations, i.e., above or below 50 per annum.

**Results:**

There were 214 participants in this study. TZ determination accuracy was 0.47 (95% CI 0.45,0.49). Accuracy for colposcopic diagnosis was 0.53 (95% CI 0.51,0.55). Decision to perform biopsies was 0.73 accurate (95% CI 0.71,0.74). Participants had 0.61 (95% CI 0.59,0.64) sensitivity and a 0.80 (95% CI 0.79,0.82) specificity for detecting high-grade lesions. Colposcopists who performed more than 50 cases were more accurate than those performed fewer across all indicators, with a higher sensitivity (0.66 vs. 0.57, *p* = 0.001) for detecting high-grade lesions.

**Conclusions:**

In medically underserved communities of China, colposcopists appear to perform poorly at TZ identification, colposcopic diagnosis, and when deciding to biopsy. Colposcopists who undertake more than 50 colposcopies each year performed better than those who perform fewer. Therefore, colposcopic practice does improve through case exposure although there is an urgent need for further pre-professional and clinical training.

## Introduction

More than 600,000 new cases of cervical cancer occur worldwide each year, with 20% occurring in mainland China [1]. China is also one of few countries experiencing an increase in cervical cancer incidence [2]. However, China has proactively heeded the World Health Organization’s call to expedite the elimination of cervical cancer. This call established ambitious targets to attain by 2030, including a 70% screening coverage and a 90% treatment coverage [3]. Colposcopy has consistently held a pivotal role in diagnosing precancerous lesions among individuals with abnormal screening outcomes, serving as a crucial guide for treatment. Therefore, the proficiency of colposcopists is a key factor in determining the overall effectiveness of cervical cancer prevention and control initiatives.

An extensive meta-analysis assessing the diagnostic capabilities of Chinese colposcopists revealed an average agreement of 68.35% when comparing colposcopic findings to histopathology results [4]. Agreement in tertiary hospitals was 70.22% compared to 61.43% agreement in primary hospitals. However, it is important to acknowledge that this meta-analysis involved more tertiary hospitals, and research into colposcopic practice in medically underserved communities remains scarce. Additionally, the aforementioned study did not assess other key competencies, such as the determination of the transformation zone (TZ) and decision to perform biopsies. These aspects represent key component of colposcopists’ skillset that warrant further scrutiny.

In medically underserved communities in China, many doctors who perform colposcopies are either obstetricians and gynecologists or in some instances midwives who may not have received systematic colposcopy training. Additionally, exposure to clinical cases is often limited, especially to high-grade cases. Although operational guidelines and quality control standards have been published by official organizations, it has been reported that practitioners find these standards difficult to follow [5]. These factors combined contribute to the inadequate capabilities of colposcopists in these areas. This study aims to quantitatively assess colposcopists’ competencies in medically underserved communities across mainland China. The findings will provide insights to improve competencies and a baseline from which to measure improvements.

## Materials and methods

### Study design and population

The recruitment announcement was published on- and off-line. Competence testing was conducted to assess colposcopists’ capabilities in medically underserved communities of China in September 2022. Primary colposcopists in primary and secondary hospitals in medically underserved communities were eligible to participate. Medically underserved communities are defined as specific populations that have a notable shortage of primary healthcare services or otherwise face unmet healthcare needs [6]. This study was approved by the Institutional Review Board in the Chinese Academy of Medical Sciences and Peking Union Medical College (Reference: CAMS & PUMC-IEC-2022-022). All participants were required to provide informed consent before participating.

### Procedure

Sociodemographic data were collected and included age, gender, ethnicity, education level, hospital level, and number of annual colposcopic examinations. The test comprised 20 cases and was designed to assess colposcopists’ competencies. Participants were provided with a series of time-stamped colposcopic images, which included an initial image along with a minimum of four additional images stained with acetic acid. Alongside these images, participants were provided colposcopic indications, according to clinical practice requirements. After thorough examination and analysis of the information provided, participants were asked to determine TZ type (either I/II or III), to provide colposcopic diagnosis (either normal/benign, low-grade squamous intraepithelial lesion [LSIL], or high-grade squamous intraepithelial lesion or worse [HSIL+]), and to decide whether or not to perform biopsies.

Cases were categorized as either normal/benign, LSIL and HSIL+ based on biopsies taken during initial colposcopy. These determinations were confirmed by an expert panel. The expert panel comprised three specialist colposcopists, each possessing over 20 years of experience at interpreting colposcopic images and evaluating more than 500 cases annually. All images underwent separate assessments by experts, and in cases of disagreement, a multilateral meeting of independent assessors was held to decide. Decisions to biopsy were taken in accordance with the ASCCP (American Society for Colposcopy and Cervical Pathology) Colposcopy Standards [7]. Patients with positive biopsies were classified according to the most severe pathological diagnosis, while cases with negative or without biopsies were graded through colposcopic consensus. The ‘ground truth’ of TZs was formulated by the expert panel in accordance with established guidelines. The TZ was categorized as type I when the entire TZ, including all upper limits, was located on the ectocervix. Type II and Type III involve endocervical components. In Type II, the upper limits of the TZ can be observed using specific devices. If the upper limits were only partially visible or completely invisible, it was classified as type III.

### Outcomes

Primary outcomes were competency indicators of all responses to the 20 cases provided. Indicators included overall accuracy rates for TZ, colposcopic diagnosis, and decision to perform biopsies, as well as accuracy rates for TZ Type I/II and Type III, colposcopic diagnosed benign/normal, LSIL, and HSIL+. Analysis included rates for over diagnoses, missed diagnoses, excessive biopsies, and missed biopsies. Diagnostic performance metrics included sensitivity, specificity, positive predictive value [PPV] and negative predictive value [NPV] for HSIL+ cases.

Participants were categorized according to the number of colposcopies performed per year. 50 colposcopies was set as threshold since the European Federation for Colposcopy (EFC) has set a minimum case load of 50 colposcopies per year [8].

### Statistical analysis

Sample size was determined using the binominal distribution formula. Colposcopic diagnoses has been reported to be 61% accurate in Chinese primary hospitals [4]. Therefore, with a confidence level of 90% and a 5% acceptable margin of error, the minimum required sample size was calculated to be 156. Sociodemographic characteristics are presented as simple numbers and percentages. The accuracy rate, over/missed rate for biopsies/diagnoses, and the diagnostic performance metrics (including sensitivity, specificity, PPV and NPV) are reported as means with corresponding 95% confidence intervals (CI). Accuracy rates between subgroups were compared using a standard *t*-test and diagnostic performance metrics were compared using a Chi-square [2] test. Statistical analysis was conducted using Stata (version 17.0) and R (version 5.3.0). The threshold for statistical significance was set at *p* < 0.05.

## Results

Social demographic characteristics are provided in Table 1. 214 colposcopists from 116 primary or secondary hospitals across 19 provinces in China were recruited. The average age was 43.44 years (SD = 8.20), with 42.06% (*n* = 90) in the 40–49 age group. Almost all participants were women (98.13%; *n* = 210). Most held bachelor’s degrees (84.11%; *n* = 180) and 92.99% worked in secondary hospitals (*n* = 199). 55.61% (*n* = 119) performed no more than 50 colposcopies per year.

**Table 1:** Sociodemographic characteristics of colposcopists

Characteristics	N (%)
Age group, years	
< 40	72 (33.64)
40–49	90 (42.06)
≥ 50	52 (24.30)
Gender	
Male	4 (1.87)
Female	210 (98.13)
Ethnicity	
Han	198 (92.52)
Others	16 (7.48)
Education level	
College degree or below	27 (12.62)
Bachelor degree	180 (84.11)
Master degree or above	7 (3.27)
Hospital level	
Primary	15 (7.01)
Secondary	199 (92.99)
Annual number of colposcopies	
≤ 50	119 (55.61)
> 50	95 (44.39)

Table 2 provides detailed information on accuracy rates for TZ, colposcopic diagnosis, and biopsy decisions. Participants who performed more than 50 colposcopies annually outperformed those who performed no more than 50 cases across all three factors. For TZ identification, accuracy rates were as follows: 0.47 (95% CI 0.45,0.49) overall, 0.45 (95% CI 0.42,0.48) for “≤50” group and 0.49 (95% CI 0.46,0.53) for “>50” group (*p* = 0.034). Accuracy was only 0.19 (95% CI 0.15, 0.23) for the “≤50” group identifying type III TZ.

**Table 2:** Accuracy rates for transformation zone, colposcopic diagnosis, and biopsy

	All participants	Annual colposcopy examination number		
		≤ 50	> 50	P
**Transformation Zone**				
Overall accuracy rate	0.47 (0.45, 0.49)	0.45 (0.42, 0.48)	0.49 (0.46, 0.53)	0.034
Accuracy rate for Type I/II	0.57 (0.54, 0.61)	0.56 (0.52, 0.60)	0.59 (0.54, 0.64)	0.163
Accuracy rate for Type III	0.22 (0.19, 0.25)	0.19 (0.15, 0.23)	0.26 (0.22, 0.31)	0.015
**Colposcopic Diagnosis**				
Overall accuracy rate	0.53 (0.51, 0.55)	0.50 (0.48, 0.53)	0.56 (0.53, 0.59)	0.005
Accuracy rate for Benign/normal	0.44 (0.40, 0.48)	0.42 (0.37, 0.48)	0.47 (0.41, 0.53)	0.138
Accuracy rate for LSIL	0.54 (0.51, 0.58)	0.54 (0.49, 0.58)	0.55 (0.50, 0.60)	0.346
Accuracy rate for HSIL+	0.59 (0.56, 0.63)	0.54 (0.49, 0.59)	0.65 (0.60, 0.71)	0.001
Over diagnosis rate	0.22 (0.21, 0.24)	0.22 (0.19, 0.24)	0.23 (0.21, 0.25)	0.213
Miss diagnosis rate	0.15 (0.14, 0.17)	0.16 (0.15, 0.18)	0.14 (0.12, 0.16)	0.027
**Biopsy**				
Accuracy rate	0.73 (0.71, 0.74)	0.71 (0.69, 0.73)	0.75 (0.72, 0.77)	0.013
Over biopsy rate	0.09 (0.07, 0.10)	0.09 (0.07, 0.10)	0.08 (0.07, 0.10)	0.269
Miss biopsy rate	0.15 (0.13, 0.16)	0.16 (0.14, 0.18)	0.14 (0.11, 0.16)	0.070

Data are presented as means with 95% confidence intervals

*Abbreviations* LSIL, Low-grade squamous intraepithelial lesion; HSIL+, High-grade squamous intraepithelial lesion or worse

Overall, participants achieved an accuracy rate of 0.53 (95% CI 0.51,0.55) for colposcopic diagnosis. Accuracy was 0.56 (95% CI 0.53, 0.59) for “>50” group and 0.50 (95% CI 0.48, 0.53) for “≤50” group, respectively (*p* = 0.005). Also, compared to the “≤50” group, the “>50” group had a lower rate of misdiagnosis (0.14 vs. 0.16, *p* = 0.027) and superior HSIL + assessment skills (0.65 vs. 0.54, *p* = 0.001). In terms of decision to biopsy, the overall level of accuracy was 0.73 (95% CI 0.71,0.74). The “>50” group had a higher level of accuracy compared to “≤50” group (0.75 vs. 0.71, *p* = 0.013).

Sensitivity, specificity, PPV and NPV were selected to assess diagnostic performances of colposcopists to detect HSIL+ lesions. Overall, sensitivity was 0.61 (95% CI 0.59,0.64), specificity was 0.80 (95% CI 0.79,0.82), PPV was 0.58 (95% CI 0.55,0.61), and NPV was 0.82 (95% CI 0.81,0.84). Compared to the “≤50” group, the “>50” group operated with higher sensitivity (0.66 vs. 0.57, *p* = 0.001) and an improved NPV (0.84 vs. 0.81, *p* = 0.032). Detailed information can be found in Table 3.

**Table 3:** Diagnostic performance for detecting HSIL+

	All participants	Annual colposcopy examination number		
		≤ 50	> 50	p
Sensitivity	0.61 (0.59, 0.64)	0.57 (0.54, 0.61)	0.66 (0.62, 0.70)	0.001
Specificity	0.80 (0.79, 0.82)	0.80 (0.78, 0.82)	0.80 (0.78, 0.83)	0.984
PPV	0.58 (0.55, 0.61)	0.56 (0.53, 0.60)	0.60 (0.56, 0.64)	0.172
NPV	0.82 (0.81, 0.84)	0.81 (0.79, 0.83)	0.84 (0.82, 0.86)	0.032

Data are presented as means with 95% confidence intervals

*Abbreviations* HSIL+, High-grade squamous intraepithelial lesion or worse; PPV, positive predictive value; NPV, negative predictive value

Overview responses from 214 colposcopists with clinical features for cases are presented in Table 4. The age of cases ranged from 25 to 51. Among them, six were diagnosed as normal/benign, eight as LSIL, and six as HSIL+. The cytology results included eight cases with no intraepithelial lesion or malignancy (NILM), five with atypical squamous cells of undetermined significance (ASC-US), two with atypical cells cannot exclude high-grade intraepithelial lesion (ASC-H), four with LSIL and one with HSIL. Five cases were HPV-negative, others were positive for at least one HPV type.

**Table 4:** Clinical features and response distribution for 20 test cases

Case	Age	Cytology	HPV Typing	Colposcopic Diagnosis	Response Distribution			Accuracy Rate
					Normal/Benign	LSIL	HSIL+	
1	32	LSIL	43	Normal/Benign	0.27	0.61	0.02	0.27
2	28	NILM	52	Normal/Benign	0.31	0.58	0.03	0.31
3	45	NILM	Negative	Normal/Benign	0.46	0.32	0.16	0.46
4	30	NILM	Negative	Normal/Benign	0.49	0.33	0.10	0.49
5	39	NILM	Negative	Normal/Benign	0.50	0.31	0.14	0.50
6	44	NILM	Negative	Normal/Benign	0.64	0.21	0.04	0.64
7	28	LSIL	33, 66	LSIL	0.01	0.41	0.53	0.41
8	33	ASC-US	33	LSIL	0.01	0.41	0.53	0.41
9	42	LSIL	16, 33, 51	LSIL	0.03	0.60	0.32	0.60
10	37	ASC-US	52	LSIL	0.07	0.69	0.16	0.69
11	35	NILM	33	LSIL	0.32	0.52	0.09	0.52
12	25	NILM	51	LSIL	0.28	0.55	0.08	0.55
13	32	ASC-US	Negative	LSIL	0.21	0.57	0.15	0.57
14	28	NILM	18	LSIL	0.17	0.59	0.21	0.59
15	31	ASC-US	16	HSIL+	0.07	0.57	0.33	0.33
16	27	ASC-US	33	HSIL+	0.03	0.38	0.56	0.56
17	50	ASC-H	33	HSIL+	0.04	0.33	0.59	0.59
18	41	HSIL	58	HSIL+	0.07	0.21	0.65	0.65
19	51	ASC-H	52	HSIL+	0.04	0.25	0.67	0.67
20	25	LSIL	16, 35	HSIL+	0.02	0.23	0.73	0.73

*Abbreviations* NILM, No intraepithelial lesion or malignancy; ASC-US, Atypical Squamous Cells of Undetermined Significance; ASC-H, Atypical cells cannot exclude high-grade squamous intraepithelial lesion; LSIL, Low-grade squamous intraepithelial lesion; HSIL(+), High-grade squamous intraepithelial lesion (or worse)

For normal/benign cases, the accuracy rates ranged from 0.27 to 0.64. 21–61% were misdiagnosed as LSIL, and 4–16% were misdiagnosed as HSIL+. When regrading cases categorized as LSIL, accuracy rates varied from 0.41 to 0.69. Additionally, between 1 and 32% were under-diagnosed as normal/benign, while 8–53% were over-diagnosed as HSIL+. For cases with HSIL+ lesions, the accuracy rate ranged from 0.33 to 0.73. 21–57% were misdiagnosed as LSIL, and 2–7% were misdiagnosed as normal/benign.

## Discussion

This study examined the clinical competencies of 214 colposcopists in underserved communities in China. Overall accuracy when determining TZ was 0.47 (95% CI 0.45,0.49). Overall diagnostic accuracy was 0.53 (95% CI 0.51,0.55), with 0.61 (95% CI 0.59,0.64) sensitivity and 0.80 (95% CI 0.79,0.82) specificity in detecting HSIL+. The accuracy of decisions to biopsy was 0.73 (95% CI 0.71,0.74). Compared with colposcopists who perform at least 50 colposcopies per year, those who perform fewer were less accurate at determining the TZ, and at colposcopic diagnosis, and decision to biopsy. These results highlight substantial problems in underserved communities of China which necessitates extensive further training for aspiring and practising junior colposcopists in these communities.

Our sample of colposcopists only achieved 0.47 accuracy in determining TZ types, with a 0.22 accuracy specifically for type III TZ. TZ is the epithelium between original squamocolumnar junction (SCJ) and new SCJ, which is susceptible to HPV infection and where squamous cervical cancer originates [9]. Therefore, determining TZ types helps to accurately diagnose lesions and for planning the excision range. Another study conducted in Europe reported 0.55 accuracy in TZ types [10], which was higher than we observed in this study. However, that study did not provide the distribution of TZ types or accuracy in relation to determining type. This study adds to this evidence-base highlighting significantly lower accuracy in determining type III TZ compared to type I/II TZ. Poor determination of type III TZ can lead to inadequate examinations of the cervical canal, resulting in missed endocervical lesion diagnoses. These missed diagnoses may be related to increased patient discomfort and perhaps an unwillingness (or lack of confidence) to commence a more thorough examination. That said, this finding is confirmed by Wei et al. [11] who observed a lower sensitivity for detecting HSIL+ in women with type III TZ compared to type I/II TZ. Colposcopists (like all clinicians) gain confidence *with* practice and it is not unreasonable to suggest there may be a need to enhance training to ensure aspiring and junior colposcopists are more confident examining patients while communicating during potentially painful physical examinations.

Overall agreement between colposcopists’ diagnoses and standardized determinations was 0.53, with a 0.61 sensitivity and 0.80 specificity in detecting HSIL+. According to a meta-analysis of Chinese primary hospitals [4], overall agreement between colposcopists and histopathology is approximately 0.61, with sensitivity and specificity for detecting HSIL+ of 0.62 and 0.91, respectively. Findings from more developed areas of China are slightly higher than those observed in this study. Accurately identifying HSIL+ is clinically important because HSIL is the action threshold for immediate treatment [12]. Although, there are a number of complexities in the process, for example there are overlapping features, sampling errors, coexisting infections, and observer variability. The colposcopists involved in this study had higher accuracy in diagnosing HSIL+ compared to other lesions, though they only achieved 0.59. Participants who performed no more than 50 colposcopies were also less accurate at diagnosing HSIL+ cases compared to those who performed more than 50 cases per year.

The identification of LSIL and normal/benign cases was also relatively low, and the rate of overdiagnosis was higher than the rate of missed diagnoses. This provides some very necessary insights into the psychology of both junior and senior colposcopists, and has implications for both training and practice. At present, even senior colposcopists in underserved communities may utilize biopsies as the *default* assessment as this is the gold standard diagnostic test. However, biopsy should be based on colposcopy-detected lesions not as a result of practitioner uncertainties since this decision comes with a number of complications such as pain, bleeding, increased infection and scarring [13]. Colposcopists lacking diagnostic capabilities often resort to biopsies or even more aggressive diagnostic procedures involving cervix excision to establish a diagnosis, which causes unnecessary physical and psychological harm to patients. This has obvious implications not least for artificial intelligence which can calculate probabilities and risk almost immediately to assist colposcopists.

Detailed analysis of each case revealed that poor diagnostic performance was due to a lack of familiarity with standard colposcopic features. Difficulty in distinguishing between normal/benign and LSIL lesions appears to be mainly due to a failure to differentiate between metaplastic squamous epithelium and thin acetowhite epithelium, especially when there is eversion of the columnar epithelium or condylomatoid lesions present. Whereas, confusion between LSIL and HSIL cases appears to be predominantly caused by a difficulty in differentiating between thin/translucent acetowhitening and thick/dense acetowhitening. In which case, considering the borders of acetowhiteness and vascular patterns could help distinguish between the two [13]. All of above indicate that recognition of colposcopic features which are fundamental are perhaps not yet core skills for colposcopists in underserved communities. It should be noted, that improving skills cannot be achieved entirely through theoretical teaching because there is a need to operate a colpscope while simultaneously interpreting (and managing) patient sensations. Therefore, training should find a balance between theory and practice.

Traditional in-person colposcopy training has limited access to cases and is restricted by time and space, which necessitates an innovative training platform. The digital education tool for colposcopy developed by Chen et al. incorporates image-based cases and comprehensive theoretical colposcopic knowledge, showing good performance at improving colposcopic competencies and confidence [14]. Traditional training is difficult to develop and maintain in resource-limited settings owing to the high cost of venues, personnel, and transportation [15, 16]. Digital platforms could therefore supplement traditional approaches, enabling a larger number of colposcopists to be trained in an efficient and low-cost manner, thereby making the training more scalable and sustainable. In addition, such tool provides valuable case resources and expert-led instructions that are difficult to access during daily practice, which could help enhance colposcopy service capabilities in low-resource areas and narrow the gap with resource-rich regions.

In addition to training, the competences of colposcopists in underserved areas could be enhanced using AI-assisted technologies. These technologies often rely on a large number of high-quality images curated and even annotated by experts which means that AI aggregates extensive experience accumulated over a long period. Therefore, AI has the potential to assist both less experienced and experienced physicians. Recently, a Colposcopic Artificial Intelligence Auxiliary Diagnostic System (CAIADS), developed by Chinese researchers, was found to have superior diagnostic sensitivity and an enhanced ability to predict biopsy sites compared to colposcopists [17, 18]. This raises questions about human-AI interactions because if technologies such as CAIADS can improve diagnostic accuracy and biopsy efficiency, it is necessary to embed a machine learning based reinforcement system which will require human decisions. This could reduce the subjectivity in traditional colposcopy procedures to some extent and enhance the repeatability of diagnoses. Therefore, deploying such a tool in resource-constrained settings may also improve colposcopists’ competencies considered in this study.

While we assessed colposcopists’ competencies in underserved communities in China, and provided an understanding of their abilities to identify TZ, make diagnoses, and take decisions to perform biopsies, this study had some drawbacks. Even though participants came from over 100 primary or secondary hospitals across 19 provinces in mainland China, the sample was relatively small. This may have caused sampling bias which may have provided an inaccurate representation. We know nothing about those who declined to participate and they may have done so for a number of reasons. Perhaps, they were simply too busy or perhaps they lacked confidence to participate which also has implications for practice in these communities. In addition, the online format of image-based testing differs from clinical reality, and there may be deviations in distribution of lesion severity compared to real-world scenarios. However, the cases included do represent a broad range and were not synthesized. Future research of this type should include more cases which may have both similarities and dissimilarities. Although, this study raised a number of additional questions about colposcopy practice in underserved communities in China, which need to be addressed for the well-being of women in these areas.

In conclusion, we observed generally poor abilities in TZ identification, colposcopic diagnosis, and biopsy determination of primary colposcopists in underserved communities of China. More experienced colposcopists, who perform more than 50 colposcopies annually are more competent than those who perform fewer than 50. This study provides a baseline to measure improvement although there is an urgent need to embed mandatory colposcopy training to ensure more case exposure for aspiring practitioners and professionals in underserved communities of China.

## Data Availability

The datasets used and/or analysed during the current study are available from the corresponding author on reasonable request.
